# A new UHPLC-MS/MS method for the screening of urinary oligosaccharides expands the detection of storage disorders

**DOI:** 10.1186/s13023-020-01662-8

**Published:** 2021-01-09

**Authors:** Michela Semeraro, Elisa Sacchetti, Federica Deodato, Turgay Coşkun, Incilay Lay, Giulio Catesini, Giorgia Olivieri, Cristiano Rizzo, Sara Boenzi, Carlo Dionisi-Vici

**Affiliations:** 1 Division of Metabolism and Metabolic Diseases Research Unit, Bambino Gesù Children’s Hospital, IRCCS, Viale San Paolo 15, 00146 Rome, Italy; 2 Metabolism Unit, Department of Pediatrics, Faculty of Medicine, Hacettepe University, Ankara, Turkey; 3 Department of Medical Biochemistry and Hacettepe University Hospitals Clinical Pathology Laboratory, Faculty of Medicine, Hacettepe University, Ankara, Turkey

**Keywords:** Oligosaccharides, Storage disorders, Pompe disease, Autophagy, Danon disease, Vici syndrome, Yunis-varon syndrome

## Abstract

**Background:**

Oligosaccharidoses are storage disorders due to enzymatic defects involved in the breakdown of the oligosaccharidic component of glycosylated proteins. The defect cause the accumulation of oligosaccharides (OS) and, depending on the lacking enzyme, results in characteristic profiles which are helpful for the diagnosis. We developed a new tandem mass spectrometry method for the screening of urinary OS which was applied to identify a large panel of storage disorders.

**Methods:**

The method was set-up in urine and dried urine spots (DUS). Samples were analysed, without derivatization and using maltoheptaose as internal standard, by UHPLC-MS/MS with MRM acquisition of target OS transitions, including Glc4, the biomarker of Pompe disease. The chromatographic run was < 30 min. Samples from patients with known storage disorders were used for clinical validation.

**Results:**

The method allowed to confirm the diagnosis of oligosaccharidoses (sialidosis, α-/β-mannosidosis, fucosidosis, aspartylglucosaminuria) and of GM1 and GM2 (Sandhoff type) gangliosidosis, by detecting specific OS profiles. In other storage disorders (mucolipidosis II and III, mucopolysaccharidosis type IVB) the analyisis revealed abnormal OS excretion with non-specific profiles. Besides Pompe disease, the tetrasaccharide Glc4 was increased also in disorders of autophagy (Vici syndrome, Yunis-Varon syndrome, and Danon disease) presenting cardiomuscular involvement with glycogen storage. Overall, results showed a clear separation between patients and controls, both in urine and in DUS.

**Conclusion:**

This new UHPLC/MS-MS method, which is suitable for rapid and easy screening of OS in urine and DUS, expands the detection of storage disorders from oligosaccharidoses to other diseases, including the novel category of inherited disorders of autophagy.

## Background

The new classification of storage disorders includes nine disease categories (i.e. oligosaccharidoses, mucolipidoses, mucopolysaccharidoses, sphingolipidoses, neuronal ceroid lipofuscinosis, disorders of lysosomal cholesterol metabolism, disorders of lysosomal transport or sorting, disorders of lysosomal protein degradation, and the recently identified inherited disorders of autophagy) and accounts for over 65 different inherited disorders [1]. Oligosaccharidoses are due to defects of lysosomal enzymes involved in the catabolic pathway for the breakdown of the oligosaccharidic component of glycosylated proteins [2]. The glycosidic groups, composed of fucose, mannose, sialic acid, galactose, and N-acetylglucosamine residues, to form glycoproteins are either N-linked (to asparagine) or O-linked (to serine or threonine) [3]. Enzymatic defects of this catabolic pathway cause the accumulation of oligosaccharides (OS) and, depending on the lacking enzyme, result in characteristic profiles of urinary OS, which are helpful for the diagnosis [4]. The different oligosaccharidoses share common clinical features, which include facial dymorphisms, dysostosis multiplex, hepato/splenomegaly, developmental delay and neurological signs, making difficult the differential diagnosis [2]. Biochemically, OS analysis is the first step for the diagnosis of oligosaccharidoses [5]. The initial methods to analyze urinary OS were based on thin layer chromatography (TLC), but this assay has limited analytical sensitivity and specificity due to interfering compounds derived, especially in early infancy, from the diet or from medications [5–7].

A more powerful tool for the analysis of OS has been provided thanks to the introduction in diagnostic laboratories of mass spectrometry (MS), which allows to characterize the different OS species through specific multiple reaction monitoring (MRM) transitions. The analysis with a triple quadrupole after sample derivatization enabled the detection of characteristic OS profiles in the urine of patients with different types of oligosaccharidoses and with other storage disorders, and was also suitable for prenatal diagnosis in amniotic fluid [3, 8, 9]. Other methods, utilizing capillary high performance anion-exchange chromatography mass spectrometry (HPAEC) or matrix-assisted laser desorption ionization time-of-flight (MALDI-TOF/TOF), with or without sample derivatization, have been developed for structural studies and for disease screening [10, 12]. Piraud et al. adapted a MALDI-TOF/TOF based method [12] to a triple quadrupole analyzer, and built a powerful technique suitable in diagnostic laboratory for the screening in urine of a large number of oligosaccharidoses and for prenatal diagnosis in amniotic fluid [4]. More recently, an ultra-high performance liquid chromatography mass spectrometry (UHPLC-MS/MS) method for urinary OS analysis requiring sample derivatization has been reported [13]. The rapid evolution of all these techniques has potentially expanded the list of identifiable diseases to other storage disorders which share with oligosaccharidoses an abnormal excretion of compounds related to this catabolic pathway [3, 4, 8–13]. Furthermore, in Pompe disease, a glycogen storage disorder due to deficiency of the lysosomal enzyme acid α-glucosidase, the profile of urinary OS by TLC analysis shows the presence of a large band which was characterized by UHPLC-MS/MS analysis as the tetrasaccharide 6-α-D-glucopyranosyl-maltotriose (Glc α 1-6Glc α 1-4Glc α 1-4Glc, designed as Glc4) [14, 15]. More recently, Glc4 has been suggested as a target biomarker for diagnosis, monitoring disease progression and to evaluate the response to enzyme replacement therapy in Pompe disease [16, 17]. Indeed, an abnormal OS profile by TLC analysis [18, 19] as been reported in Yunis-Varon syndrome, an inherited disorder of autophagy, a novel disease group listed among storage disorders [1].

In this study, we report a new UHPLC-MS/MS method, not requiring sample derivatization, for the screening of OS in urine and in dried urine spots (DUS) which was applied to identify a large panel of storage disorders.

## Methods

### Samples collection and complience with ethic guidelines

Controls’ and patients’ urines and DUS samples were collected after obtaining informed consent. The work has been carried out in accordance with “The Code of Ethics of the World Medical Association (Declaration of Helsinki) for experiments involving humans”; “Uniform Requirements for manuscripts submitted to Biomedical journals” published by the International Committee of Medical Journal Editors. Samples were obtained from patients—followed by the Division of Metabolism, Bambino Gesù Childrens Hospital in Rome, Italy and by the Department of Pediatrics, Metabolism Unit, Hacettepe University, Ankara, Turkey—with a confirmed diagnosis made through enzymatic and/or genetic analysis or from positive quality controls provided by the European Research Network for evaluation and improvement of screening, Diagnosis and treatment of Inherited disorders of Metabolism (ERNDIM). Urine samples (n = 42) were obtained from 27 patients, age 7 months- 17 years, affected by the following storage disorders: sialidosis (n = 1), α-mannosidosis (n = 2), β-mannosidosis (n = 1), fucosidosis (n = 3), aspartylglucosaminuria (n = 1), GM1 gangliosidosis (n = 14), GM2 gangliosidosis (n = 6), mucolipidosis type II (n = 3) and mucolipidosis type III (n = 2), Pompe disease (n = 4), Vici syndrome (n = 4), Danon disease (n = 1). Samples were kept frozen at − 20 °C until analysis. DUS samples (n = 29) were obtained from 25 patients, age 1–18 years, affected by the following storage disorders: sialidosis (n = 1), α-mannosidosis (n = 3), β-mannosidosis (n = 1), fucosidosis (n = 3), aspartylglucosaminuria (n = 1), GM1 gangliosidosis (n = 4), GM2 gangliosidosis (n = 1), mucolipidosis type II (n = 1), mucolipidosis type III (n = 2), mucopolysaccharidosis type IVb (n = 5), Pompe disease (n = 2), Vici syndrome (n = 3) and Yunis-Varon syndrome (n = 1), Danon disease (n = 1). The control urine (n = 75), age 2 months- 17 years and DUS (n = 12), age 1 months -18 years, samples were obtained from healthy subjects referred for routine urine laboratory analysis. Urine samples were kept at − 20 °C and DUS at room temperature until analysis.

A positive internal quality control (iQC) mix, containing an equal part of lyophilized urine from patients affected by sialidosis, α-mannosidosis, β-mannosidosis, fucosidosis, aspartylglucosaminuria, GM1 gangliosidosis, GM2 gangliosidosis, Pompe disease provided by ERNDIM, was reconstituted, aliquoted and stored at − 20 °C.

### Urine samples treatment

Urine samples were ultra-filtered with an Amicon Ultra filter 0.5 mL 3 K 96 PK (Merck KGaA, Darmstadt, Germany) and centrifuged for 9 min at 13.000 rpm. Ultra-filtered urine were diluted with milli-Q water (Milli-Q Advantage A10 System, Merckmillipore, Merck KGaA, Darmstadt, Germany) to obtain a final creatinine concentration of 1 mM. The creatinine concentration was determined with the Jaffé method. Samples with a creatinine concentration < 1 mmol/L were not diluted and were adjusted at a creatinine concentration of 1 mM after the analysis.

Fifty μL of normalized ultra-filtered urine were mixed in a glass tube with 20 µL of the internal standard (IS) working solution [maltoheptaose (Glc7) 170 µmol/L dissolved in H_2_O], 130 μL of reconstitution buffer [37.5% acetonitrile (ACN)/H_2_O containing 0.02% formic acid (FOA) (v/v)], and vortexed.

### Dried urine spot (DUS) samples treatment

To prepare DUS samples 2 ml of urine were spotted, both in the laboratory and in the clinic, on the entire absorbing part of the card commonly used for newborn screening (EBF Eastern Business Forms, INC., SC, US) and dried at room temperature for at least 2 h. DUS were shredded and mixed in deionized water for 30 min for the extraction. The entire absorbing part of the newborn screening card was used for the extraction. The extract was then diluted with Milli-Q water to obtain a creatinine concentration of 1 mM. The extract creatinine concentration was determined with the Jaffé method. Extracts with a creatinine concentration < 1 mmol/L were not diluted and were adjusted at a creatinine concentration of 1 mM after the analysis. Fifty μL of the normalized extract from DUS were mixed in a glass tube with 20 µl of the IS working solution, 130 μL of reconstitution buffer, and vortexed.

### UHPLC-MS/MS

Five μL of the finally urine or DUS samples respectively prepared as described in “Urine samples treatment” and “Dried urine spot (DUS) samples treatment” sections, were injected in the UHPLC system Agilent 1290 Infinity II (Agilent Technologies, CA, US) for the chromatographic analysis. The chromatography was performed with a Luna Omega SUGAR 100 column Å, 150 × 2.1 mm (Phenomenex, CA, US) at a flow rate of 0.5 mL/min. The mobile phase was a mixture of ammonium formate (Sigma-Aldrich Steinheim, Germany) 5 mM + 0.05% (v/v) FOA (> 96% purity, reagent grade Sigma-Aldrich Steinheim, Germany) dissolved in water (A) and ammonium formate 5 mM + 0.05% (v/v) FOA dissolved in ACN (≥ 99.9% purity for HPLC, gradient grade, Sigma-Aldrich Steinheim, Germany)/ water 90/10 (B). The gradient program is showed in Table 1. The total run time was 27.50 min (15.50 min for detection and 12.00 min for column reconditioning) at a controlled column temperature of 40 °C.

**Table 1:** Optimized UHPLC conditions for the separation of oligosaccharide components

Time (min)	A%	B%	Flow rate (ml/min)
0	5	95	0.5
5	60	40	0.5
13.30	60	40	0.5
13.50	30	70	0.5
15.50	30	70	0.5
17.50	5	95	0.5
27.50	5	95	0.5

A: ammonium formate 5 mM + 0.05% (v/v) formic acid in water; B: ammonium formate 5 mM + 0.05% (v/v) formic acid in acetonitrile/water 90/10

The UHPLC system was interfaced to a triple quadrupole 4500 SCIEX QTrap (AB Sciex, MA, US) equipped with a turbo ion spray source heated at 400 °C. Nitrogen was used as curtain and collision gas. Common MS/MS parameters expressed in arbitrary units were the following: curtain gas (CUR), 20; ion source gas 1 (GS1), 20; ion source gas 2 (GS2), 20; collision-activated dissociation gas (CAD), 9; temperature (TEM), 400. In positive mode MS/MS parameters were: ion spray voltage (ISV), 5.500 V; entrance potential (EP), 8 V; cell exit potential (CXP), 10 V; in negative mode MS/MS parameters were: ISV, − 4.500 V; EP, − 8 V; CXP, − 10 V.

### Oligosaccharides detection

MRM was used for spectra acquisition in positive and negative modes, switching the polarity within a single run. Data acquisition and chromatographic peak integration were conducted using the Analyst software (version 1.7 with HotFix 2, ®2017 AB SCIEX, Canada), using Glc7 as IS. For each sample, ratios of peak area/Glc7 was calculated for single MRM transitions. For each MRM, the median (50th percentile) of at least 10 control samples was calculated and results were expressed as multiple of the medians (MoM).

The positive iQC was treated with the same procedure of samples and added to each batch series to control chromatography quality and the sensitivity of the tandem mass spectrometer.

## Results

### Chromatography and mass spectra

**Fig. 1:**
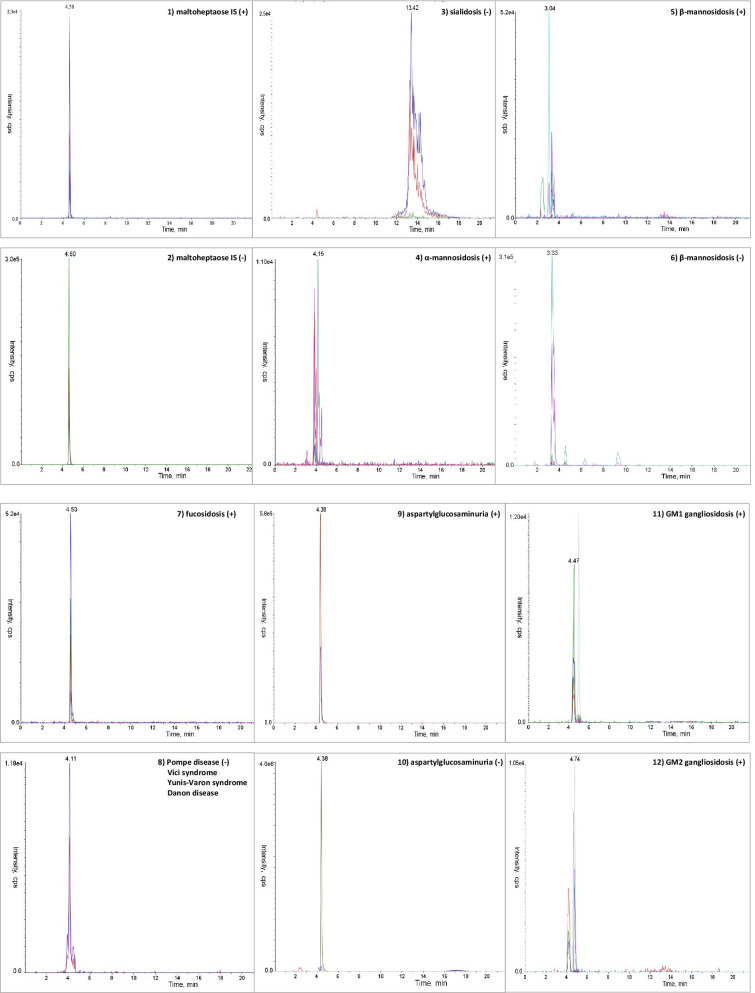
Chromatographic profiles of the examined storage disordes and of the internal standard (IS). The characteristic MRM transitions, in positive (+) and negative (−) modes, for each disorder are marked with different colours. Panel A: (1) extract ion chromatogram (XIC) of the seven positive MRM transitions characteristic for the IS maltoheptaose Glc7; (2) XIC of the seven negative MRM transitions characteristic for Glc7; (3) XIC of the three negative MRM transitions characteristic for sialidosis; (4) XIC of the eight positive MRM transitions characteristic for α-mannosidosis; (5) XIC of the nine positive MRM transitions characteristic for β-mannosidosis; (6) XIC of the eight negative MRM transitions characteristic for β-mannosidosis. Panel B: (7) XIC of the six positive MRM transitions characteristic for fucosidosis; (8) XIC of the two negative MRM transitions characteristic for Pompe disease, Vici syndrome, Yunis-Varon syndrome, and Danon disease; (9) XIC of the five positive MRM transitions characteristic for aspartylglucosaminuria; (10) XIC of the ten negative MRM transitions characteristic for aspartylglucosaminuria; (11) XIC of the five positive MRM transitions characteristic for GM1 gangliosidosis; (12) XIC of the seven positive MRM transitions characteristic for GM2 gangliosidosis

Figure 1 shows the extract ion chromatogram (XIC) of a selection of storage disorders (sialidosis, α- and β-mannosidosis, fucosidosis, aspartylglucosaminuria, GM1 and GM2 gangliosidosis, Pompe disease, Vici syndrome, Yunis-Varon syndrome and Danon disease) presenting a characteristic OS profile [4, 15].

### Assay validation

For each MRM transition, linearity was estimated with calibration curves created using three samples at different urine concentrations and calculating the correlation coefficient (R^2^). The differently concentration samples were prepared by mixing and vortexing in a glass tube, as follow: 50 μL of normalized ultra-filtered urine, 20 µL of the IS working solution and 130 μL of reconstitution buffer; 100 μL of normalized ultra-filtered urine, 20 µl of the IS working solution and 80 μL of reconstitution buffer; 150 μL of normalized ultra-filtered urine, 20 µL of the IS working solution and 30 μL of reconstitution buffer. In fresh urine, the R^2^ ranged from 0.8039 to 0.9999 in the positive mode and from 0.7270 to 0.9999 in the negative mode. In DUS, R^2^ ranged from 0.7701 to 0.9996 in the positive mode and from 0.8340 to 0.9995 in the negative mode.

For each MRM transition, the intra-day precision, expressed as CV%, was assessed by injecting and analyzing 10 times in the same run 5 µL of the finally urine or DUS samples respectively prepared as described in “Urine samples treatment” and “Dried urine spot (DUS) samples treatment” sections; the inter-day precision, expressed as CV%, was assessed by injecting and analyzing 10 times for three different days 5 µL of the finally urine or DUS samples prepared respectively as described in “Urine samples treatment” and “Dried urine spot (DUS) samples treatment” sections.

The intra-day precision ranged from 6 to 24% in the positive and negative mode in fresh urine, from 5 to 25% in the positive mode and from 2 to 24% in the negative mode in DUS. The inter-day precision ranged from 9 to 25% in the positive mode and from 8 to 23% in the negative mode in fresh urine, from 7 to 26% in the positive mode and from 5 to 24% in the negative mode in DUS. Validation data of linearity, intra-day and inter-day precision in urine and DUS are reported in Additional file 1: A and B.

### Detection of oligosaccharides in storage disorders

The study allowed to confirm the diagnosis in all disorders presenting known OS profiles. Additional file 2: A and B illustrate the MoM results obtained from all analyzed samples. Each sample was considered positive for a specific disorder when the MoMs of all qualifying and quantifying transitions were increased compared to controls at least 5 times for fresh urine and 2 times for DUS. Some disorders presented with a specific OS profile while in other diseases, the analysis revealed abnormal OS excretion but not-specific profiles. Overall, DUS samples showed lower background interferences when compared to urine samples.

#### Disorders with a specific OS profile

**Fig. 2:**
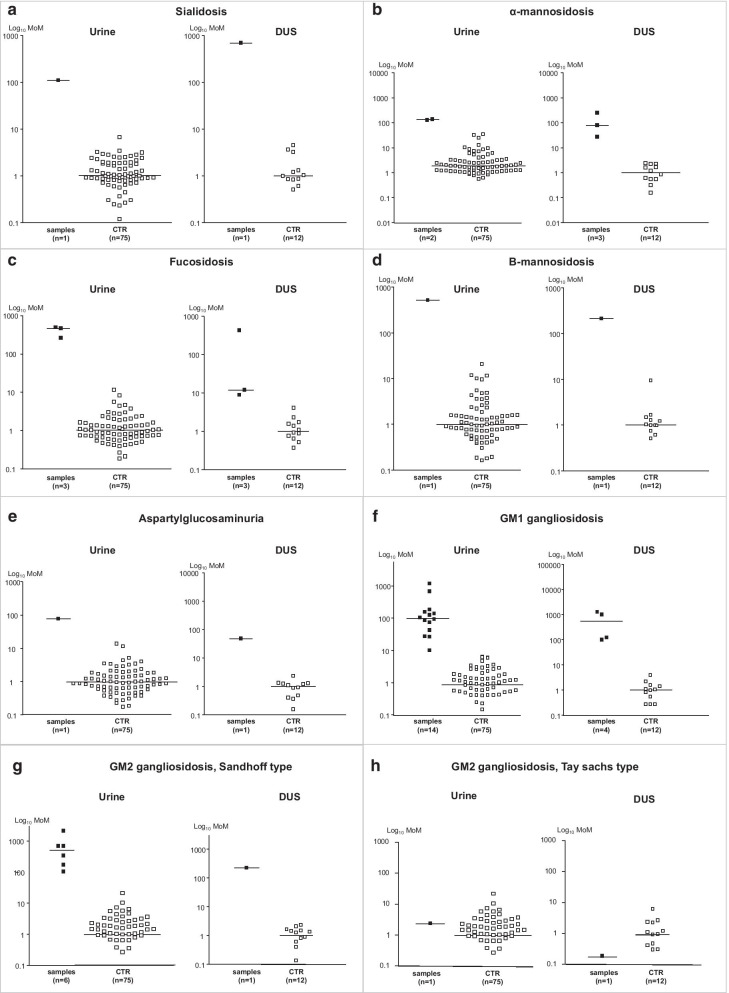
Scatter charts of the most characteristic transitions in disorders showing specific OS profiles. The figure shows for each storage disorders the most characteristic MRM transitions in urine and DUS in comparison to controls: **a** sialidosis: transition 1200.4 > 1099.4 of the sialyl-OS NeuAc-Hex3HexNAc2; **b** α-mannosidosis: transition 568.2 > 347.2 of the mannosyl-OS NeuAc-Hex3HexNAc2; **c** fucosidosis: transition 504.2 > 289.2 of the fucosyl-OS Fuc-HexNAc-Asn; **d** β-mannosidosis: transition 406 > 244 of Hex-HexNAc and derivatives; **e** aspartylglucosaminuria: transition 520.2 > 305.2 of the GlcN-Asn + glycoasparaginyl-OS Hex-HexNAc-Asn; **f** GM1 gangliosidosis: transition 933.5 > 388.3 of the galactosyl-OS Hex3-HexNAc2; **g** GM2 gangliosidosis, Sandhoff type: transition 1136.3 > 933.4 of the N-acetylgalactosaminyl-OS Hex3-H; **h** GM2 gangliosidosis, Tay-Sachs type lacking the increase of the transition 1136.3 > 933.4 of the N-acetylgalactosaminyl-OS Hex3-HexNAc3 as seen in GM2 Sandhoff type

Figure 2 shows the scatter charts of the most characteristic transitions in disorders showing specific OS profiles [4].

##### Sialidosis

Three negative MRM transitions were selected as the most characteristic for the sialyl-OS NeuAc-Hex3-HexNAc2 and one was used for quantitative analysis. We analyzed 1 urine and 1 DUS sample from one patient and both quantitative and qualitative MRMs were highly increased compared to controls, with 102 fold elevation in urine and 668 fold in DUS.

##### α-Mannosidosis

Eight positive MRM transitions for the three mannosyl-OS, Hex2-HexNAc, Hex3-HexNAc and Hex4-HexNAc were selected as the most characteristic, and 4 were used for quantitative analysis. We analyzed 2 urine samples and 3 DUS obtained from 2 patients and all quantitative and qualitative MRMs were highly increased compared to controls, with a 110–118 fold elevation in urines and 29–245 fold in DUS for the most characteristic transition.

##### β-Mannosidosis

Nine positive and eight negative MRM transitions for Hex-HexNAc and its derivatives were selected as the most characteristic, and 5 were used for quantitative analysis. We analyzed 1 urine sample and 1 DUS from a patient and all quantitative and qualitative MRMs were highly increased compared to controls, with 720 fold elevation in urine and 155 fold in DUS for the most characteristic transition.

##### Fucosidosis

Six positive MRM transitions for the two fucosyl-OS, Fuc-HexNAc-Asn and Fuc-HexNAc2-Hex3, were selected as the most characteristic, and 3 were used for quantitative analysis. We studied 4 patients and analyzed 3 urine samples and 3 DUS and all quantitative and qualitative MRMs were highly increased compared to controls, with 220–470 fold elevation in urine and 152–373 fold in DUS for the most characteristic transition.

##### Aspartylglucosaminuria

Five positive MRM transitions for aspartylglucosamine GlcN-Asn and the glycoasparaginyl-OS Hex-HexNAc-Asn and ten negative MRMs transitions for aspartylglucosamine GlcN-Asn and the glycoasparaginyl-OS NeuAc-Hex-HexNAc-Asn were selected as the most characteristic, and 3 positive and 3 negative MRMs were used for quantitative analysis. We studied 1 patient with aspartylglucosaminuria and analyzed 1 urine sample and 1 DUS and all quantitative and qualitative MRMs were highly increased compared to controls, with 71 fold elevation in urine and 80 fold in DUS for the most characteristic transition.

##### GM1 gangliosidosis

Five positive MRM transitions for the two galactosyl-OS, Hex3-HexNAc2 and Hex5-HexNAc3 were selected as the most characteristic, and 3 were used for quantitative analysis. We studied 7 patients and analyzed 14 urine samples and 4 DUS and all quantitative and qualitative MRMs were highly increased compared to controls, with 9–1160 fold elevation in urine and 69–883 fold in DUS for the most characteristic transition.

##### GM2 gangliosidosis (Sandhoff & Tay-Sachs diseases)

Seven positive MRM transitions for the three N-acetylglucosaminyl-OS, Hex2-HexNAc2, Hex3-HexNAc3 and Hex3-HexNAc4 were selected as the most characteristic of GM2 gangliosidosis O variant (Sandhoff disease) and 4 were used for quantitative analysis. We studied 4 Sandhoff patients and analyzed 6 urine and 1 DUS. In all samples, quantitative and qualitative MRMs were highly increased compared to controls, with 99–2102 fold elevation in urine and 79 fold in DUS for the most characteristic transition.

In the 3 patients with GM2 gangliosidosis B variant (Tay-Sachs disease) the OS analysis in urine and DUS did not display abnormalities.

**Fig. 3:**
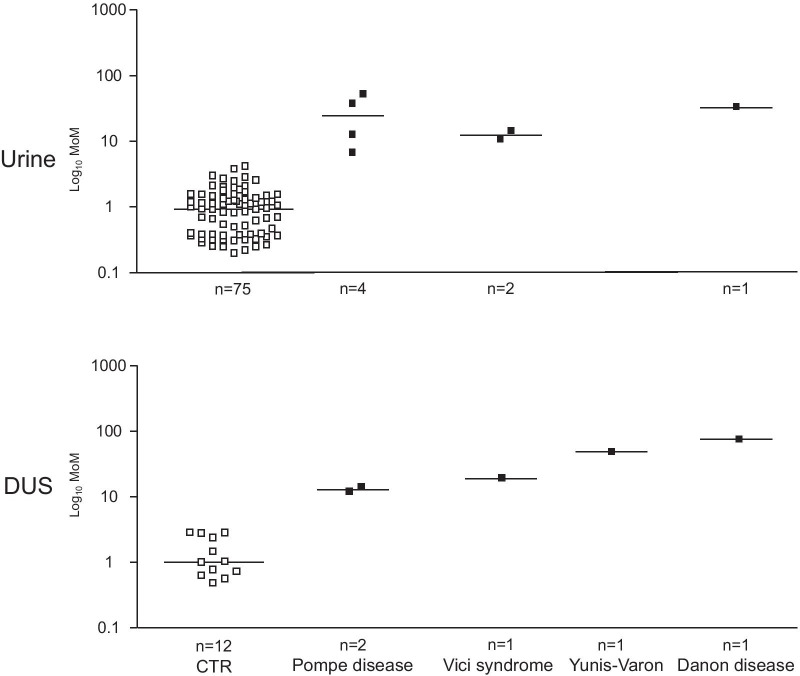
Scatter charts of disorders presenting an increased excretion of Glc4 which included, besides Pompe disease, also the autophagy related disorders Vici and Yunis-Varon syndromes, and Danon disease. The figure shows the scatter charts in urine (top panel) and DUS (lower panel) of the transition 665 > 179 of the tetrasaccharide 6-α-D-glucopyranosyl-maltotriose (Glc4) in Pompe disease, Vici syndrome, Yunis-Varon syndrome, and Danon disease in comparison to controls

Figure 3 shows the scatter charts of disorders presenting an increased excretion of Glc4 which included, besides Pompe disease, also the autophagy related disorders Vici and Yunis-Varon syndromes, and Danon disease.

##### Pompe disease (glycogen storage disease type II)

Two negative MRM transitions of the tetrasaccharide Glc4 and of its isomer maltotetraose (M4) were selected as the most characteristic. We studied 3 untreated patients, two with infantile-onset and one with late-onset Pompe disease, and analyzed 4 urine and 2 DUS. Glc4 was increased compared to controls, with a 10–72 fold elevation in urine and 11–13 fold in DUS, respectively. Lower levels were detected in the patient with milder phenotype.

##### Vici syndrome

We studied 3 patients, one with the classic severe clinical picture and two sibs with a milder phenotype, and analyzed 4 urines and 3 DUS. The patient with the most severe variant showed an 21–28 fold increase of Glc4 in urine and an 18 fold increase in DUS, while the two sibs with the less severe variant displayed only minor OS abnormalities both in urine and DUS and no elevation of Glc4.

##### Yunis-Varon syndrome

The DUS sample from the patient with Yunis-Varon syndrome showed a 51 fold elevation of Glc4.

##### Danon disease (Glycogen storage disease type II B)

We studied 1 patient with Danon disease and analyzed 1 urine and 1 DUS. Glc4 was increased compared to controls, with a 45 fold elevation in urine and 75 fold in DUS.

#### Disorders with a not-specific OS profile

##### Mucolipidosis type II, and type III

We studied 2 patients with mucolipidosis type II and analyzed 3 urines and 1 DUS. In all urine samples OS analysis revealed mixed non specific profiles with 8/8 positive transitions of α-mannosidosis and 5/5 positive transitions of GM1 gangliosidosis.

We studied 2 patients with mucolipidosis type III and analyzed 2 urines and 2 DUS. In urine 5/5 positive MRM transitions of GM1 gangliosidosis were variably increased, however to a lesser extent than in GM1.

##### Mucopolysaccharidosis IVB

DUS from 5 patients were available and all samples displayed increased levels of Glc4 however to a lesser extent than the positivity threshold.

## Discussion

The diagnosis of storage disorders is often challenging due the presence of common clinical features and great variability in symptoms, and requires a complex approach which includes the analysis of target biomarkers in biological fluids, the measurement of enzymatic activities in leukocytes and/or fibroblasts and the confirmatory diagnosis by mutation analysis [20]. The availability of novel and specific therapies for a large number of diseases, including oligosaccharidoses, has increased the medical demand of reliable diagnostic techniques to offer precise and timely diagnosis. For this purpose, we developed a new UHPLC-MS/MS method for the screening of OS in urine and in DUS. The method was applied to screen known samples of a large panel of storage disorders and allowed to confirm the expected diagnoses (Table 2).

**Table 2:** List of storage disorders screened with the UHPLC-MS/MS method

Storage disorders	Enzyme/protein deficiency	Gene	MIM number	Characteristic oligosaccharides
*Oligosaccharidoses*				
Sialidosis	α-d-neuraminidase	*NEU1*	608272	Sialyl-OS
α-Mannosidosis	α-d-mannosidase	*MAN2B1*	609458	Mannosyl-OS
β-Mannosidosis	β-d-mannosidase	*MANBA*	609489	Hex-HexNAc and derivates
Fucosidosis	α-l-fucosidase	*FUCA1*	612280	Fucosyl-OS
Aspatylglucosaminuria	N-aspartyl-β-glucosaminidase	*AGA*	613228	GlcN-Asn + glycoasparaginyl-OS
*Sphingolipidoses*				
GM1-gangliosidosis	β-d-galactosidase	*GLB1*	611458	Galactosyl-OS
GM2-gangliosidosis O variant (Sandhoff)	Hexosaminidase A and B	*HEXB*	606873	N-acetylgalacto saminyl-OS
GM2-gangliosidosis B variant (Tay-Sachs)	Hexosaminidase A	*GM2A*	613109	No abnormalities
*Glycogen storage disorders*				
Pompe disease (Glycogenosis type II)	Acid α-glucosidase	*GAA*	606800	Glc4
*Disoders of autophagy*				
Vici syndrome	EPG5	*EPG5*	615068	Glc4
Yunis-Varon syndrome	FIG4	*FIG4*	609390	Glc4
	VAC 14	*VAC14*	604632	
Danon disease (Glycogenosis type IIb)	LAMP2	*LAMP2*	309060	Glc4
*Mucolipidoses*				
Mucolipidosis type II & III	N-acetylglucosamine-1-P-transferase	*GNPTAB*	607840	Non-specific abnormalities
*Mucopolisaccharidoses*				
Mucopolysaccharidosis IVB	β-d-galactosidase	*GLB1*	611458	Non-specific abnormalities

All examined oligosaccharidoses (i.e. sialidosis, α-/β-mannosidosis, fucosidosis, aspartylglucosaminuria) and both GM1 and GM2 (Sandhoff type only) gangliosidosis displayed specific OS profiles, as shown by the presence of single or multiple qualifying and quantifying MRM transitions [4]. In other diseases, such as mucolipidosis type II-III, and mucopolysaccharidosis type IVB the analysis revealed abnormal (non-specific) OS excretion, with mixed patterns combining MRM transitions of different disorders. Each OS was identified through its specific MRM transitions in positive and in negative modes [4]. Given the chemical similarity, we included in the OS analysis also the negative MRM transition of Glc4, the urinary biomarker of Pompe disease [15, 17]. This implementation allowed to confirm the diagnosis of Pompe disease, with some variations in Glc4 excretion related to disease phenotypic severity.

Besides Pompe disease, Glc4 levels were markedly increased also in three disorders belonging to the autophagy machinery [21–23]. These include Vici syndrome and Yunis-Varon syndrome, two diseases characterized by a complex multisystem phenotype, and Danon disease, a disorder with clinical manifestations very similar to Pompe disease [24–28]. As seen in glycogen storage disorders, all these conditions share a cardio-muscular involvement with increased glycogen storage and variable vacuoles accumulation detectable on light microscopy at the level of skeletal and cardiac muscle,. In previous studies on Yunis-Varon syndrome, abnormalities of urinary OS by TLC analysis have been reported [18, 19]. However, given the technical limitation of this method, a precise identification of these compounds was missed in the original reports. Our UHPLC-MS/MS method confirmed a relevant increase of OS excretion in Yunis-Varon syndrome and allowed to identify Glc4 as the disease-target compound. As a novel finding, upon evaluation of samples from patients with Vici syndrome and Danon disaese we detected a striking increase of Glc4 in urine. The structural and pathogenetic similarities with Pompe disease indicate that the increased urinary excretion of Glc4 in these three autophagy-related disorders reflects the abnormal muscle glycogen breakdown [25, 26], as also seen in other muscle disorders causing glycogen storage such as GSD type 3 and 6 [15, 29]. Glc4 could therefore serve as a biomarker to screen a wider range of storage disoders, including autophagy-related diseases, presenting cardiomuscular sings with glycogen storage.

From a technical perspective, most of recently reported methods using triple quadrupole for the analysis of OS require sample derivatization with 1-phenyl-3-methyl-5-pyrazolone, making the preanalitycal phases of these methods time consuming [3, 8, 9]. Two MALDI-TOF/TOF mass spectrometry methods, lacking internal standard, and suitable for disease screening have been reported [11, 12], with one not requiring sample derivatization [12].

The advantages of our screening method for OS analysis include a short preanalytical phase, not requiring sample derivatization, the use of UHPLC-MS/MS platform, a more widely available apparatus in diagnostic laboratories than MALDI-TOF/TOF mass spectrometry, and the possibility to perform OS analysis in urine and DUS, with comparable diagnostic results, thus making simpler and easier sample shipment and storage. The chromatography was performed with a HILIC phase column, which was chosen for its ability to improve retention and selective separation of sugar-related compounds. Unlike the more commonly used aminic columns, the HILIC column showed an enhanced lifetime because of the stationary phase composed with highly robust and thermally modified fully porous particles. The gradient was optimized to obtain the elution of all compounds in about 15 min, with a residual 12 min for column reconditioning. The assay validation of the method in urine and DUS, showed that the correlation coefficient and the intra/inter-day precision of positive and negative MRM transitions were comparable in the two biological matrices. Moreover, in the control population, we noted that most of quantitative transitions showed significant differences for age below 6 month (12) versus eldest age (63) (see Additional file 3).

For each MRM, results obtained in patients and controls showed a clear separation of the two populations, both in urine and in DUS, without overlapping.

## Conclusions

This new UHPLC/MS-MS method, not requiring sample derivatized and allowing the rapid and easy detection of OS in urine and DUS, expands the screening of storage disorders from oligosaccharidoses to other diseases, including Pompe disease and the novel category of inherited disorders of autophagy causing abnormal muscle glycogen breakdown.

## Supplementary Information

**Additional file 1: Table S1a.** Validation data of positive MRM transitions; **Table S1b.** Validation data of negative MRM transitions.

**Additional file 2: Table S2a.** Results of urinary oligosaccharides analysis expressed as multiple of the median (MoM); **Table S2b.** Results of oligosaccharides analysis in DUS expressed as multiple of the median (MoM).

**Additional file 3.** Box plots showing, for the most characteristic transitions of storage disorders, significant differences in control groups for values < 6 month and > 6 month of age.

## Data Availability

Data supporting the findings of this study are available as electronic additional files.

## Abbreviations

OS: Oligosaccharides
DUS: Dried urine spots
TLC: Thin layer chromatography
MS: Mass spectrometry
MRM: Multiple reaction monitoring
HPAEC: High performance anion-exchange chromatography mass spectrometry
MALDI-TOF/TOF: Matrix-assisted laser desorption ionization time-of-flight
UHPLC-MS/MS: Ultra-high performance liquid chromatography mass spectrometry
Glc4: Tetrasaccharide 6-α-d-glucopyranosyl-maltotriose
ERNDIM: European Research Network for Evaluation and Improvement of Screening, Diagnosis and Treatment of Inherited Disorders of Metabolism
iQC: Internal quality control
IS: Internal standard
Glc7: Maltoheptaose
ACN: Acetonitrile
FOA: Formic acid
CUR: Curtain gas
GS1: Ion source gas 1
GS2: Ion source gas 2
CAD: Collision-activated dissociation gas
TEM: Temperature
ISV: Ion spray voltage
EP: Entrance potential
CXP: Cell exit potential
MoM: Multiple of the median
XIC: Extract ion chromatogram
R^2^: Correlection coefficient
