# Supplementary material for: A new UHPLC-MS/MS method for the screening of urinary oligosaccharides expands the detection of storage disorders
Source: Orphanet J Rare Dis. 2021 Jan 9;16:24. doi: 10.1186/s13023-020-01662-8 (PMC7796585; doi:10.1186/s13023-020-01662-8)
Supplement: Supplementary file 1 — Additional file 1: Table S1a. Validation data of positive MRM transitions; Table S1b. Validation data of negative MRM transitions. [file 13023_2020_1662_MOESM1_ESM.docx]

**Table S1a. Validation data of positive MRM transitions**

| **Oligosaccharidoses** | **intra-day CV%** | | **inter-day CV%** | | **Linearity ( r^2^)** | |
| --- | --- | --- | --- | --- | --- | --- |
| MRM | Urine | DUS | Urine | DUS | Urine | DUS |
| ***Galactosyl-OS*** |  |  |  |  |  |  |
| 933,5>388,3 | 6.3 | 6.6 | 10.5 | 7.3 | 0.9532 | 0.9750 |
| 933,5>550,3 | 23.4 | 17.6 | 24.1 | 18.5 | 0.9154 | 0.9640 |
| 933,5>568,3 | 18.9 | 8.4 | 21.0 | 9.6 | 0.9869 | 0.9380 |
| 933,5>712,3 | 20.7 | 11.0 | 22.0 | 15.3 | 0.9740 | 0.9012 |
| 1460,6>1095,5 | 13.1 | 5.1 | 15.8 | 7.8 | 0.9981 | 0.9830 |
| ***N-acetylgalactosaminyl-OS*** |  |  |  |  |  |  |
| 771,3>568,4 | 17.4 | 13.0 | 17.5 | 14.2 | 0.9205 | 0.9584 |
| 771,3>550,4 | 19.5 | 12.1 | 19.9 | 13.6 | 0.9950 | 0.9782 |
| 771,3>347,2 | 20.5 | 19.5 | 21.5 | 20.2 | 0.9423 | 0.9210 |
| 1136,3>933,4 | 19.0 | 20.3 | 21.3 | 21.5 | 0.8630 | 0.9990 |
| 1339,4>1136,5 | 15.7 | 22.5 | 17.6 | 24.9 | 0.8039 | 0.9996 |
| 1339,4>933,4 | 21.3 | 10.2 | 22.6 | 13.8 | 0.8942 | 0.9971 |
| 1339,4>712,3 | 18.8 | 20.6 | 19.1 | 24.1 | 0.9881 | 0.9893 |
| ***Mannosyl-OS*** |  |  |  |  |  |  |
| 568.2>244.1 | 21.4 | 17.1 | 22.6 | 23.2 | 0.9702 | 0.9112 |
| 568.2>347.2 | 19.9 | 22.2 | 20.6 | 22.8 | 0.9253 | 0.7701 |
| 568.2>365.2 | 19.9 | 20.4 | 20.7 | 23.6 | 0.9114 | 0.9384 |
| 568.2>406.3 | 23.5 | 12.0 | 23.7 | 14.7 | 0.9482 | 0.9178 |
| 568.2>467.3 | 23.4 | 25.7 | 24.5 | 26.1 | 0.8580 | 0.9918 |
| 568.2>550.3 | 16.6 | 25.1 | 18.5 | 26.8 | 0.9210 | 0.9947 |
| 730.3>509.3 | 23.4 | 12.0 | 24.8 | 13.7 | 0.9370 | 0.9691 |
| 892.4>671.4 | 21.7 | 23.9 | 23.2 | 24.5 | 0.8540 | 0.9819 |
| ***Hex-HexNAc and derivatives*** |  |  |  |  |  |  |
| 384>81 | 15.5 | 10.2 | 19.8 | 12.1 | 0.8702 | 0.8410 |
| 406>203 | 23.7 | 6.1 | 24.6 | 13.9 | 0.9750 | 0.9815 |
| 406>226 | 18.2 | 14.6 | 20.1 | 17.1 | 0.9992 | 0.9579 |
| 406>244 | 22.4 | 16.0 | 23.7 | 18.8 | 0.8723 | 0.9820 |
| 406>305 | 19.2 | 9.0 | 19.4 | 13.2 | 0.8562 | 0.9992 |
| 406>388 | 17.0 | 14.4 | 19.1 | 18.2 | 0.9780 | 0.8796 |
| 407>203 | 7.1 | 12.1 | 9.5 | 14.1 | 0.9350 | 0.9718 |
| 407>226 | 21.0 | 9.1 | 24.3 | 11.2 | 0.9660 | 0.9987 |
| 407>389 | 19.8 | 22.5 | 23.1 | 25.2 | 0.9570 | 0.9791 |
| ***Fucosyl-OS*** |  |  |  |  |  |  |
| 504.2>289.2 | 24.2 | 24.4 | 25.2 | 25.9 | 0.8392 | 0.9982 |
| 504.2>358.2 | 18.1 | 7.0 | 19.5 | 9.6 | 0.9763 | 0.9920 |
| 504.2>389.2 | 24.5 | 19.5 | 25.6 | 21.7 | 0.9850 | 0.9597 |
| 504.2>459.3 | 22.2 | 25.0 | 23.2 | 25.5 | 0.8616 | 0.9910 |
| 1079.4>933.4 | 21.8 | 10.1 | 22.7 | 11.9 | 0.9293 | 0.9598 |
| 1079.4>568.3 | 24.8 | 24.5 | 25.3 | 24.7 | 0.9999 | 0.9713 |
| ***GlcNAsn+glycoasparaginyl-OS*** |  |  |  |  |  |  |
| 336.2>204.1 | 17.8 | 22.2 | 18.5 | 23.4 | 0.9907 | 0.9952 |
| 336.2>133.1 | 18.7 | 23.6 | 19.9 | 24.6 | 0.9710 | 0.9680 |
| 336.2>126.1 | 17.8 | 15.8 | 18.4 | 18.9 | 0.9999 | 0.9890 |
| 336.2>84.0 | 17.8 | 17.9 | 18.4 | 19.1 | 0.9997 | 0.9210 |
| 520.2>305.2 | 16.1 | 13.0 | 17.7 | 17.4 | 0.9742 | 0.9780 |

**Table S1b. Validation data of negative MRM transitions**

| **Oligosaccharidoses** | **intra-day CV%** | | **inter-day CV%** | | **Linearity ( r^2^)** | |
| --- | --- | --- | --- | --- | --- | --- |
| MRM | Urine | DUS | Urine | DUS | Urine | DUS |
| ***Sialyl-OS*** |  |  |  |  |  |  |
| 1200.4>1099.4 | 12.3 | 6.2 | 13.9 | 14.2 | 0.9901 | 0.9995 |
| 1200.4>1182.4 | 12.2 | 11.9 | 12.6 | 16.2 | 0.9879 | 0.9890 |
| 1200.4>289.6 | 24.2 | 24.7 | 23.8 | 24.9 | 0.9493 | 0.9742 |
| Hex-HexNAc and derivatives |  |  |  |  |  |  |
| 418>59 | 23.3 | 3.9 | 21.4 | 8.9 | 0.7530 | 0.9912 |
| ***418>119*** | 20.7 | 3.3 | 23.9 | 8.5 | 0.8324 | 0.9693 |
| 418>179 | 11.0 | 12.1 | 15.1 | 16.8 | 0.8154 | 0.9110 |
| 418>263 | 13.4 | 15.1 | 12.6 | 16.1 | 0.8321 | 0.9750 |
| 418>281 | 9.1 | 6.4 | 10.4 | 8.2 | 0.7720 | 0.8340 |
| 480>79 | 24.5 | 2.3 | 22.4 | 5.5 | 0.9999 | 0.9993 |
| 480>97 | 17.7 | 4.2 | 18.6 | 8.9 | 0.8793 | 0.9889 |
| 545>162 | 6.8 | 7.4 | 8.5 | 9.4 | 0.7270 | 0.9412 |
| GlcNAsn+glycoasparaginyl-OS |  |  |  |  |  |  |
| ***334.0>97.0*** | 18.2 | 12.1 | 19.5 | 15.8 | 0.9753 | 0.9750 |
| 334.0>113.0 | 17.1 | 7.9 | 17.7 | 10.1 | 0.9410 | 0.9930 |
| 334.0>195.9 | 12.3 | 14.2 | 15.2 | 16.3 | 0.7710 | 0.9578 |
| 334.0>315.9 | 13.7 | 3.6 | 14.6 | 7.5 | 0.7993 | 0.9700 |
| 787.3>496.2 | 10.8 | 7.0 | 12.9 | 9.1 | 0.9987 | 0.9489 |
| 787.3>478.2 | 11.8 | 5.0 | 14.1 | 8.9 | 0.9950 | 0.9488 |
| 787.3>214.1 | 9.8 | 10.1 | 10.2 | 11.0 | 0.9971 | 0.9520 |
| 787.3>179.0 | 12.4 | 8.8 | 13.8 | 11.9 | 0.9895 | 0.9370 |
| 809.3>290.1 | 13.6 | 11.3 | 15.6 | 12.3 | 0.9966 | 0.9278 |
| ***809.3>236.3*** | 13.8 | 20.4 | 14.9 | 19.6 | 0.9995 | 0.9910 |
| Glc4-M4 |  |  |  |  |  |  |
| 665>179 | 21.0 | 14.0 | 22.6 | 23.5 | 0.9800 | 0.9298 |
| 665>161 | 23.9 | 17.9 | 21.8 | 24.7 | 0.9690 | 0.9881 |
