# Supplementary figures and images for: A new UHPLC-MS/MS method for the screening of urinary oligosaccharides expands the detection of storage disorders
Source: Orphanet J Rare Dis. 2021 Jan 9;16:24. doi: 10.1186/s13023-020-01662-8 (PMC7796585; doi:10.1186/s13023-020-01662-8)

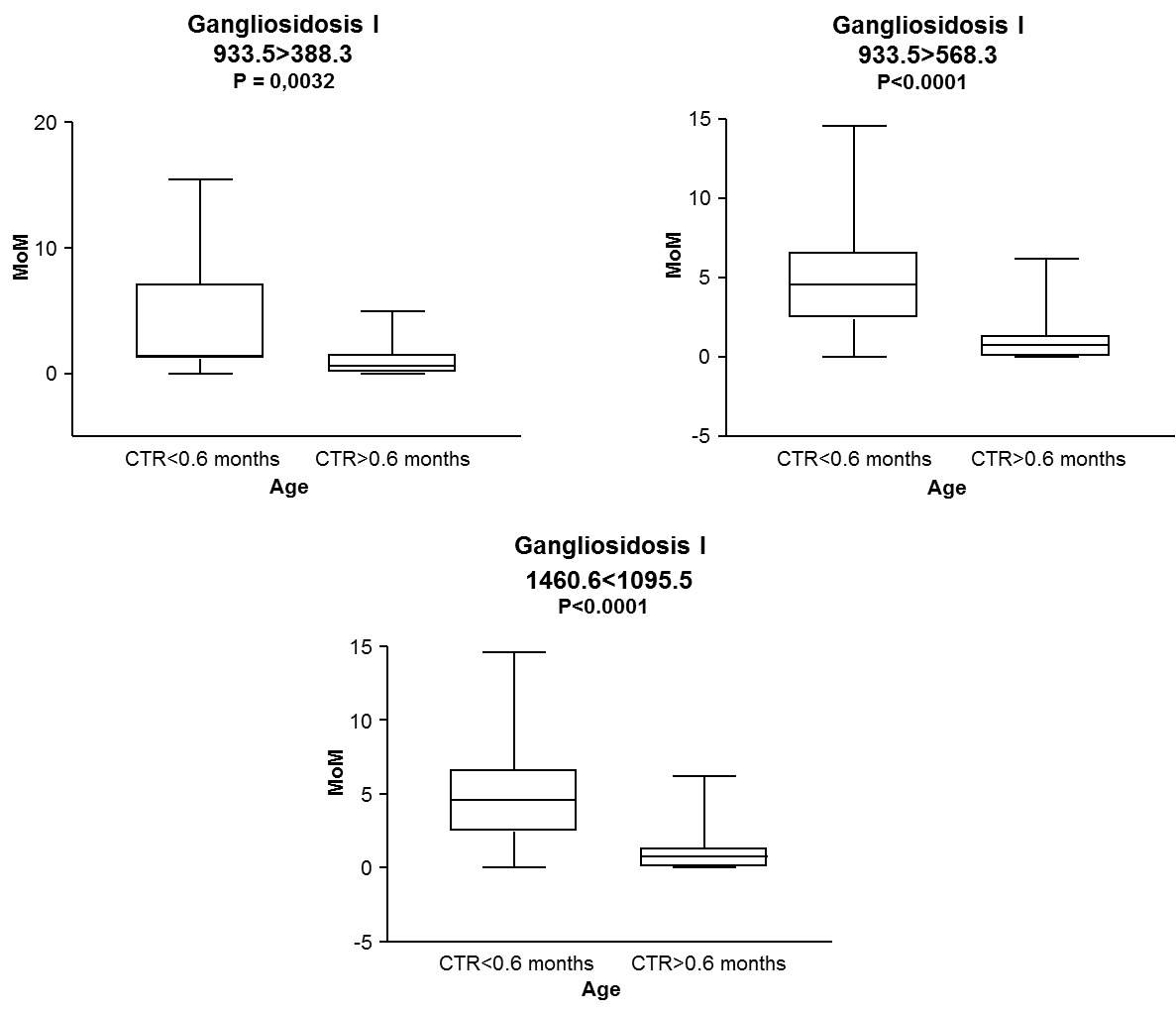


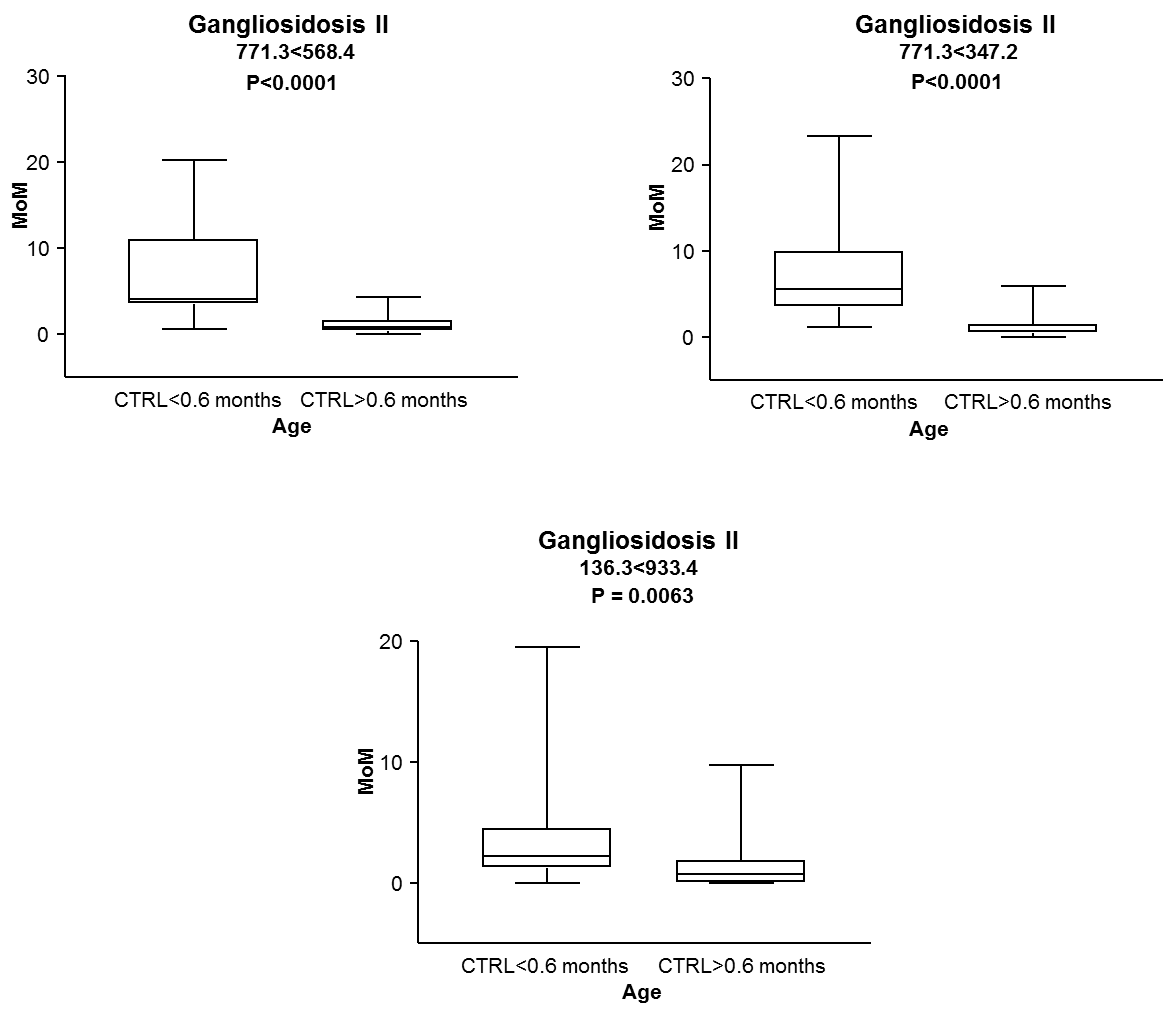


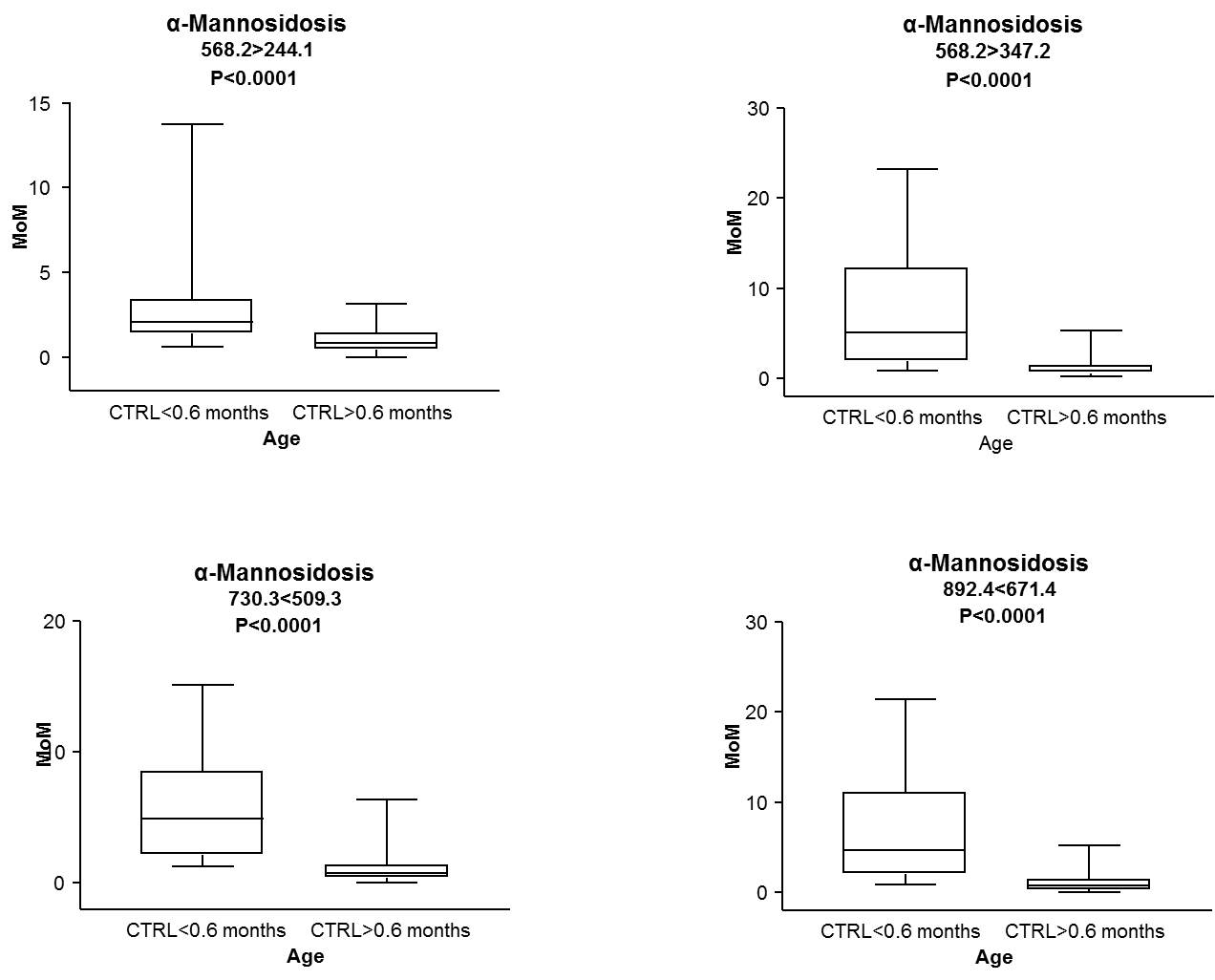


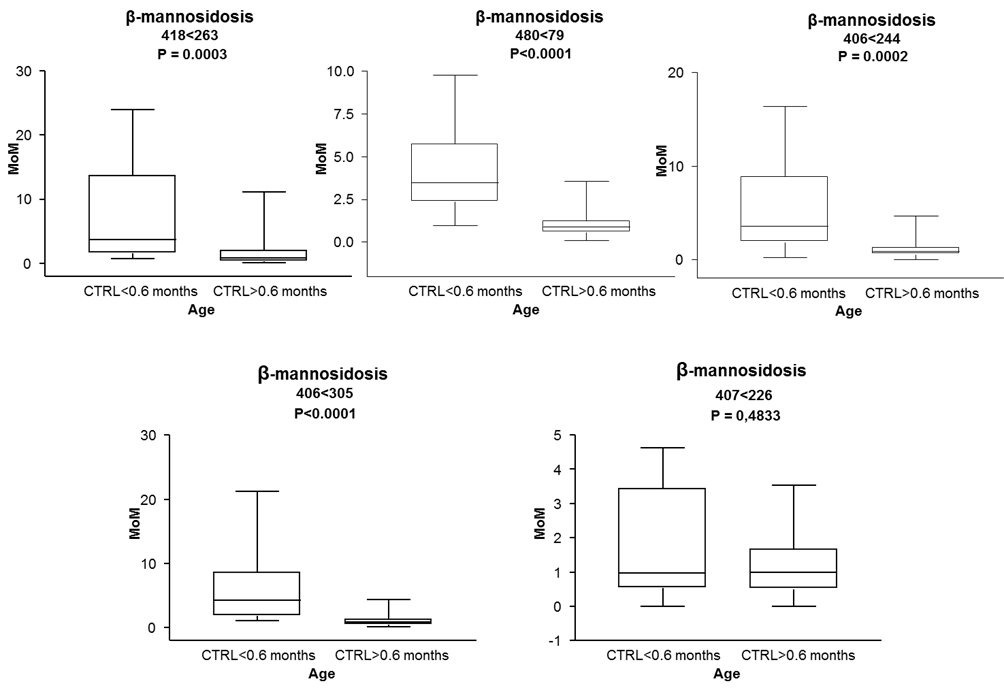


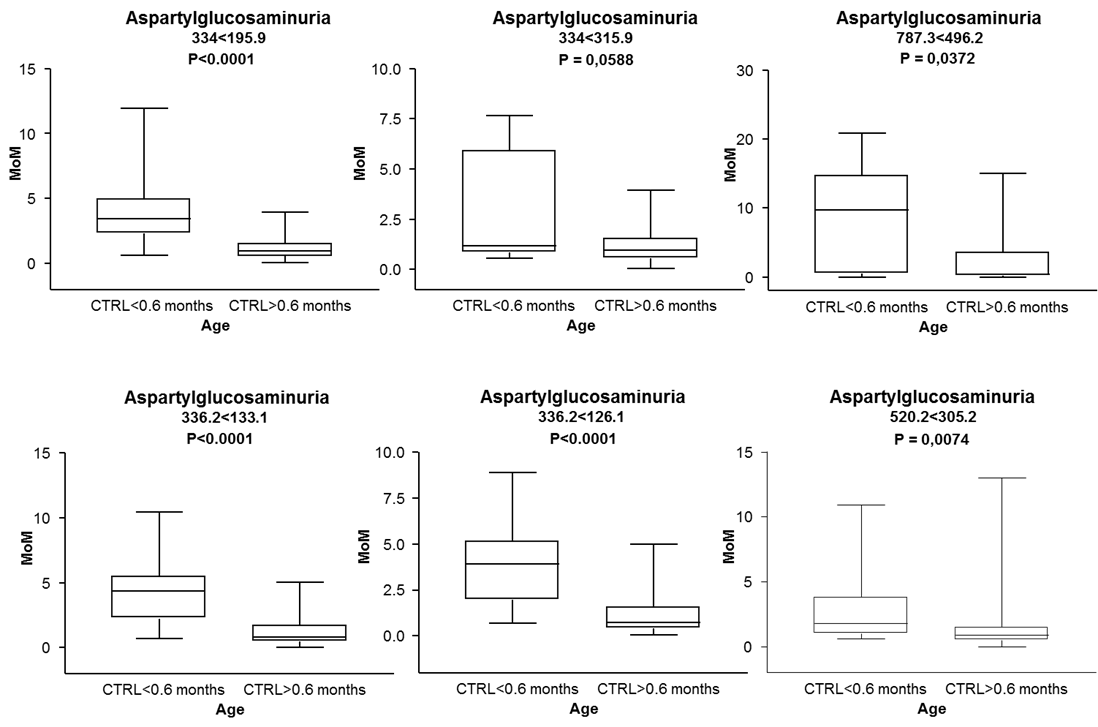


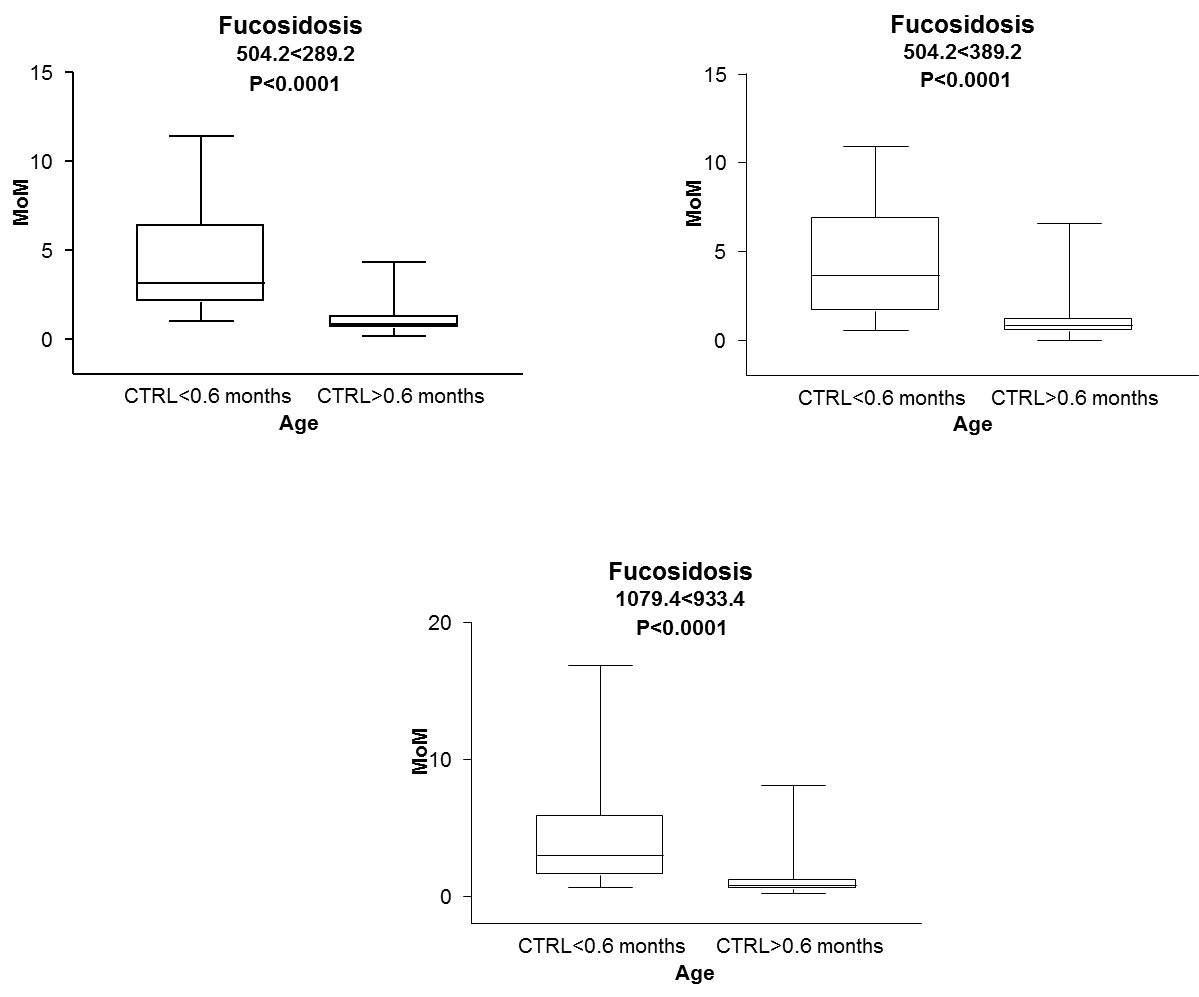


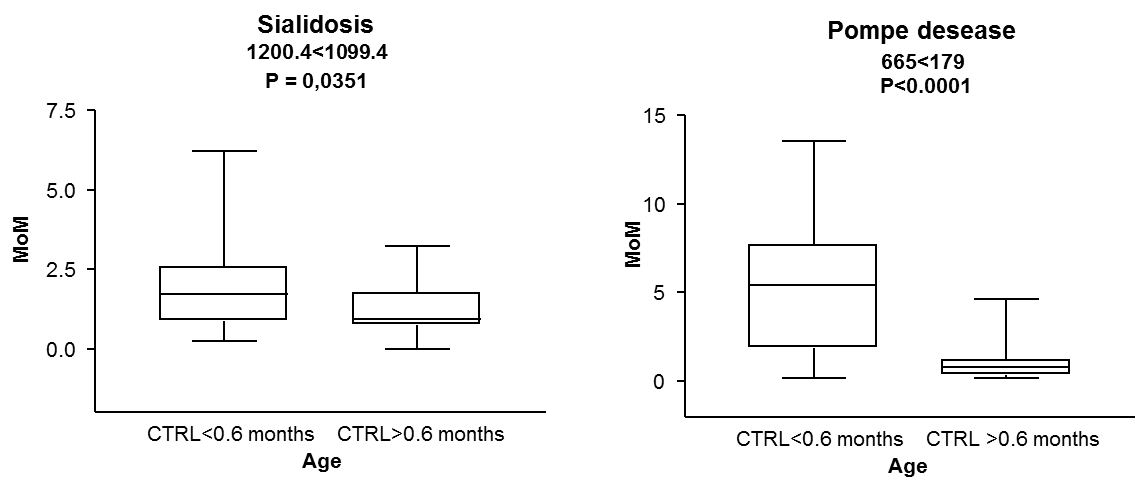

Supplement: Supplementary file 3 — Additional file 3. Box plots showing, for the most characteristic transitions of storage disorders, significant differences in control groups for values < 6 month and > 6 month of age. [file 13023_2020_1662_MOESM3_ESM.docx]
